# Clinical and Epidemiological Analysis of Children’s Urinary Tract Infections in Accordance with Antibiotic Resistance Patterns of Pathogens

**DOI:** 10.3390/jcm10225260

**Published:** 2021-11-12

**Authors:** Katarzyna Werbel, Dorota Jankowska, Anna Wasilewska, Katarzyna Taranta-Janusz

**Affiliations:** 1 Department of Pediatrics and Nephrology, Medical University of Białystok, 15-274 Białystok, Poland; katarzynawerbel@gmail.com (K.W.); annwasil@interia.pl (A.W.); 2 Department of Statistics and Medical Informatics, Medical University of Białystok, 15-295 Białystok, Poland; dorota.jankowska@umb.edu.pl

**Keywords:** antibiotic resistance, *Escherichia coli*, inflammatory markers, urinary tract infection

## Abstract

The study was conducted to analyze urinary tract infections (UTI) in children by considering epidemiology and antibiotic resistance patterns of pathogens in accordance with inflammatory parameters. The research included 525 patients who demonstrated 627 episodes of UTI. The increasing resistance of bacteria was observed over the years covered by the study (*p* < 0.001). There was a significant increase of resistance to amoxicillin with clavulanic acid (*p* = 0.001), gentamicin (*p* = 0.017) and ceftazidime (*p* = 0.0005). According to the CART method, we managed to estimate C-reactive protein (CRP), procalcitonin (PCT) and white blood cell (WBC) values, in which antibiotic sensitivity was observed. In children with CRP > 97.91 mg/L, there was a high percentage of sensitive cases to amoxicillin with clavulanic acid (87.5%). Values of WBC above 14.45 K/µL were associated with *E. coli* more sensitivity to ampicillin. 100% of children with CRP > 0.42 mg/L and PCT ≤ 6.92 ng/mL had confirmed sensitivity to cefuroxime. Concerning sensitivity to gentamicin, the most optimal cut-off point of WBC was >7.80 K/µL, while in the case of nitrofurantoin, it was CRP value > 0.11 mg/L (which was presented in 98.50% of children). These results may guide us with antibiotic therapy and help to inhibit increasing antibiotic resistance.

## 1. Introduction

Urinary tract infections (UTI) are known as one of the most common bacterial diseases in children (after respiratory tract infections). Furthermore, they are often the cause of infant and toddler hospitalization [1]. Proper management of a UTI episode is a renal scarring prophylaxis, especially when associated with congenital anomalies of the urinary tract. Renal scarring may lead to complications in adulthood, including hypertension, proteinuria, renal damage and even chronic kidney disease. Therefore, it is important to initiate an effective antibiotic therapy early. Considering the numerous reports from the USA, Canada and Europe on the growing antibiotic resistance associated with often and inadequate use of antibacterial drugs, it is justified to regularly reassess the antibiotic sensitivity of bacterial strains responsible for UTI [2,3].

Due to conditions present in childhood age, such as short urethra, possibility of contamination with fecal bacteria, higher incidence of congenital defects and continuous development of human organ systems (including the immune system), the child’s organism is more likely to develop UTI than an adult’s. UTI occurs in 2–8% of children. Infections of this system affect 0.1–1% of full–term newborns [4], while in premature infants, this percentage rises to 25% [5].

In early infancy (especially <3 months old), the male sex is more predisposed to UTI due to higher incidence of CAKUT (congenital anomalies of kidney and urinary tract), whereas in adolescence, infections are more common in girls [6,7]. The incidence of UTI differs depending on race, which in white people is almost twice as high as in the African American population [8].

The most dominant etiological factor of UTI is *Escherichia coli*, which is responsible for about 90% of infections [2,9,10]. Proteus spp. is often found in urine cultures of young male infants because of its presence under the foreskin. Other bacteria known to be etiological factors of UTI are *Pseudomonas aeruginosa*, *Klebsiella pneumoniae* and *Enterobacter* spp. [11,12]. *Pseudomonas aeruginosa*, due to its properties, is a common cause of recurrent UTI.

Incidence of UTI is more frequent in groups of children with risk factors, including anomalies of urinary tract (including vesicoureteral reflux–VUR) and dysfunctional elimination syndrome [13,14].

The discovery of first antibiotic (penicillin) is one of the biggest achievements of medicine. Over the decades, it has helped to save millions of human lives. However, the common use of antibiotics reduced the effectiveness of this type of treatment. Klein et al. [15] analyzed global consumption of antibiotics from 2000 to 2015. The research showed that the highest DDD rates (daily defined dose) per 1000 habitants in 2015 were found in Turkey, Tunisia (~50 DDD), Spain, Greece, Algeria and Romania (~35–40 DDD). The biggest increase of DDD over the years covered by the study was observed in Turkey, Tunisia and Algeria.

It is known that the process of acquiring resistance is a natural example of evolution in response to usage of antibiotics. Unfortunately, irrational antibiotic therapy accelerates this trend. Tadesse et al. [16], conducted the research, in which they found out that multi–drug resistance of *Escherichia coli* is systematically increasing. The study showed the increase of resistance from 7.2% in the years from 1950–1959 to 63.6% in the years from 2000–2002.

Laboratory tests that can be helpful in making a diagnosis are markers of inflammation, e.g., elevated concentration of C-reactive protein–CRP. High CRP values are frequently found in patients with upper urinary tract infection and CRP testing has been shown to be useful in differentiating upper and lower UTI [17]. Raised CRP values may also be found in lower UTI in smaller children and as a result, there is a risk that antibiotics may be wrongly prescribed. For valid interpretations of inflammatory markers’ results in patients with UTI, awareness of the range of values that can be expected when the infection is caused by antibiotic sensitive microorganisms would be useful.

The main aim of the study was to analyze antibiotic resistance trends over the years. We also assessed inflammatory parameters’ association with patients’ responsiveness to prescribed drugs and if it is possible to plan empiric therapy on the basis of inflammatory markers levels.

## 2. Materials and Methods

The retrospective study was conducted in 525 children hospitalized in the Department of Pediatrics and Nephrology (Children’s Clinical University Hospital of Białystok, Poland) from 2010–2017, in which we observed 627 episodes of UTI. The inclusion criterion for the study group was a positive urine culture with accompanying clinical symptoms (a negative microbiological test result was acceptable in the case of use of outpatient antibiotic therapy before collecting urine for cultures in 33 cases; in these patients, UTI episodes were diagnosed and treatment had been started by general practitioner). The exclusion criteria were insignificant bacteriuria, leukocyturia with negative urine culture (except cases with administered outpatient antibiotic therapy). From among the performed microbiological tests, a significant growth of bacteria was obtained in 590 cases. Initially, 711 cases of UTI were included in the preliminary analysis. After excluding cases that did not meet the inclusion criteria or were not eligible for the study due to the exclusion criteria, the final analysis contained 627 episodes of UTI (398 in girls and 229 in boys). 318 episodes were febrile UTIs; patients without fever were hospitalized due to other symptoms, such as vomiting or severe abdominal pain. Twenty-nine of excluded cases were asymptomatic bacteriuria. Patients with history of recurrent UTIs and with VUR grade III–V were on chemoprohylaxis (110 girls and 51 boys). Renal scars and features of nephropathy were assessed in 51 cases by performing DMSA (as a feature of nephropathy (most often reflux nephropathy), a hypofunction of kidney determined as the split kidney function <30% shown on DMSA). Participants were aged between 7 days old and almost 18 years old (331 females and 194 males). The anthropometric measurements and laboratory tests were carried out at the time of admission to the hospital. In every patient, blood laboratory tests were performed, which included white blood count (WBC), C-reactive protein and procalcitonin (PCT). Blood count was examined from K-EDTA blood (Sysmex XT-4000i, Sysmex Europe GMBH, Bornbarch, Germany), however CRP and PCT concentrations were measured in serum with a clot activator (Cobas 6000 c501, Roche Diagnostics International Ltd., Rotkreuz, Switzerland). Urine samples for urinalysis and urine culture were collected by clean–catch of midstream urine or catheterization, also in infants (the method of clean–catch of midstream urine was used in cases when parents refused to consent catheterization). The use of a urine bag was only permitted for urinalysis. After bacteria growth in the urine culture, the antibiograms were marked in order to assess sensitivity of microbes to commonly used antibiotics (ampicillin, amoxicillin with clavulanic acid, cefuroxime, ceftazidime, gentamicin, cotrimoxazole and nitrofurantoin).

The research results were statistically analyzed using the Statistica 13.3 computer program (StatSoft, Tulsa, OK, USA). In the analyzes and tests, the significance level of *p* < 0.05 was used. For the analyzed numerical features, in each of the estimated group, the normality of the distribution was assessed using the Shapiro-Wilk test. Due to the lack of normal distribution the studied groups were compared with the non-parametric Mann-Whitney U test. Additionally, the non-parametric Pearson’s Chi-square test of independence was used to assess the relationship between categorical features. The method of classification and regression trees (CART) was used to establish the optimal cut–off point for inflammatory markers, which allow to classify cases as sensitive or resistant to analyzed antibiotics.

The study was carried out after agreement of the Bioethics Committee of the Medical University of Bialystok (R-I-002/104/2018).

## 3. Results

The study included a total of 525 children hospitalized in the Department of Pediatrics and Nephrology (Children’s Clinical University Hospital of Białystok, Poland) due to UTI in years 2010–2017. Girls constituted 63.1% (331) of children covered by the study, while boys—36.9% (194). Demographic and clinical parameters of participants are shown in Table 1.

**Figure 1:**
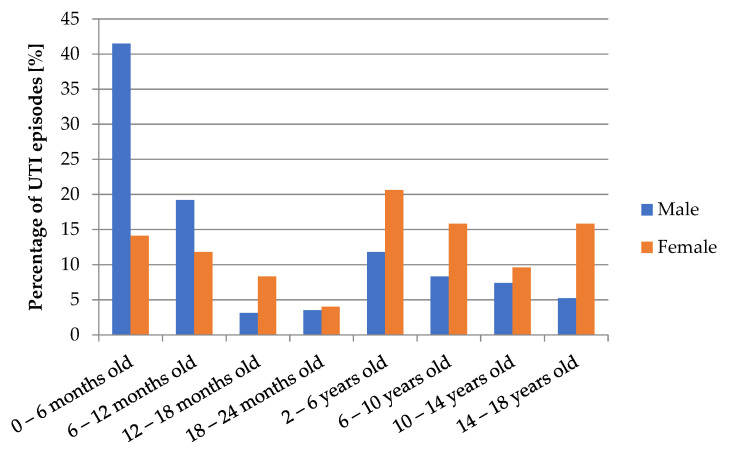
Number of UTI episodes depending on age and gender of patients.

The youngest patient developed UTI in the first week of life, while the oldest patient was 17 years and 11 months old. The median age was 2.08 years old. The age of patients with UTI differ significantly between males and females (*p* < 0.001). The analysis showed that girls have greater body weight than boys (*p* < 0.001), but there was no significant difference in BMI values (*p* = 0.12). Comparisons of concentrations of inflammatory markers showed significantly higher values of WBC in males (*p* = 0.009). Values of CRP and PCT did not differ significantly (*p* > 0.05). The diagram in Figure 1 shows the variability of UTI episodes depending on age and sex of patients. In boys, UTI was diagnosed mainly in the first year of life (with the prevalence of infections <6 months old). In girls, the distribution of UTI incidents was much more regular, with predomination of infections diagnosed after the second year of life.

In the study group, 147 patients had a history of recurrent UTIs—girls constituted 69.4% (102) and boys—30.6% (45). The recurrent UTIs were observed in 30.8% of girls and in 23.2% of boys. There was no significant dependence of sex on recurrent UTIs incidence (*p* = 0.06).

One of the risk factors of UTI is the presence of anatomical and functional abnormalities of the urinary tract. In the study group, 171 children were diagnosed with the urinary tract defect (Table 2) in which VUR was by far the most common diagnosis (92 patients).

In Table 3, the number of children with VUR depending on sex is shown. The conducted analysis showed that the gender of patient had a statistically significant dependence on the grade of VUR (*p* = 0.013). In boys, a high-grade VUR was observed more often (48.4%) than in girls (24.6%).

Renal scintigraphy (DMSA) was performed in 51 patients to assess kidney function. In 31.4% of cases features of nephropathy were found, while in 19.6%, there were renal scars.

**Figure 2:**
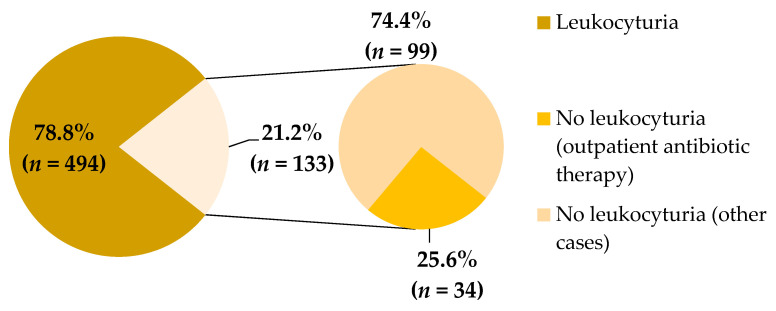
Percentage of leukocyturia in studied urine samples.

The diagram in Figure 2 presents the percentages of urine samples with or without leukocyturia. Leukocyturia was demonstrated in 78.8% of analyzed samples. The outpatient antibiotic therapy was administered in 34 cases out of 133 urine samples with no leukocyturia.

**Figure 3:**
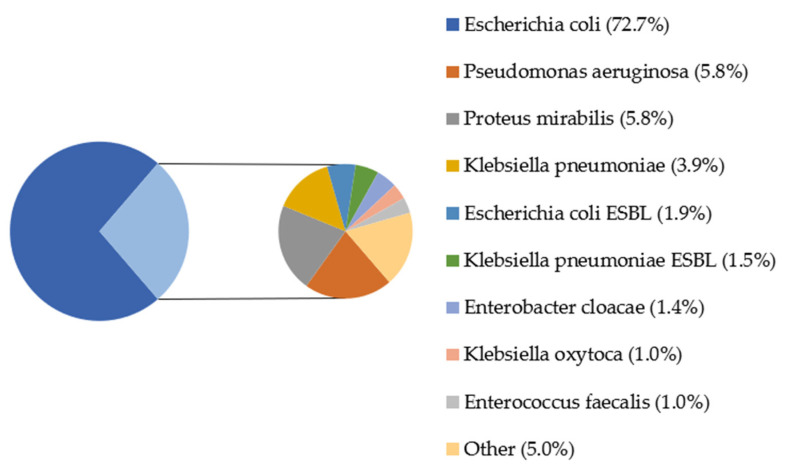
Percentage of bacterial strains in the urine cultures of patients; ESBL—extended-spectrum beta-lactamases. Other: *Proteus vulgaris* (0.68%), *Citrobacter freundii* (0.52%), *Citrobacter koseri*, *Staphylococcus saprophyticus* MSCNS, *Staphylococcus saprophyticus*, *Acinetobacter baumanii* (each 0.35%), *Enterococcus gallinarum*, *Escherichia coli* AMPc, *Staphylococcus epidermidis* MRSE, *Enterococcus faecalis* HLAR, *Providencia rettgeri*, *Streptococcus agalactiae*, *Streptococcus mitis*, *Morganellamorgani*, *Staphylococcus aureus*, *Enterobacter aerogenes*, *Staphylococcus simulans*, *Micrococcus* spp., *Staphylococcus epidermidis* MSCNS MLSB, *Staphylococcus epidermidis* (each 0.171%).

In Figure 3, there are shown percentages of microbes from urine cultures covered by the study. The most dominant bacteria in samples was *Escherichia coli* (72.7%). Other microbes found in the study were *Pseudomonas aeruginosa* (5.8%), *Proteus mirabilis* (5.8%) and *Klebsiella pneumonia* (3.9%); each of the rest of bacteria represented less than 2% of the total.

**Figure 4:**
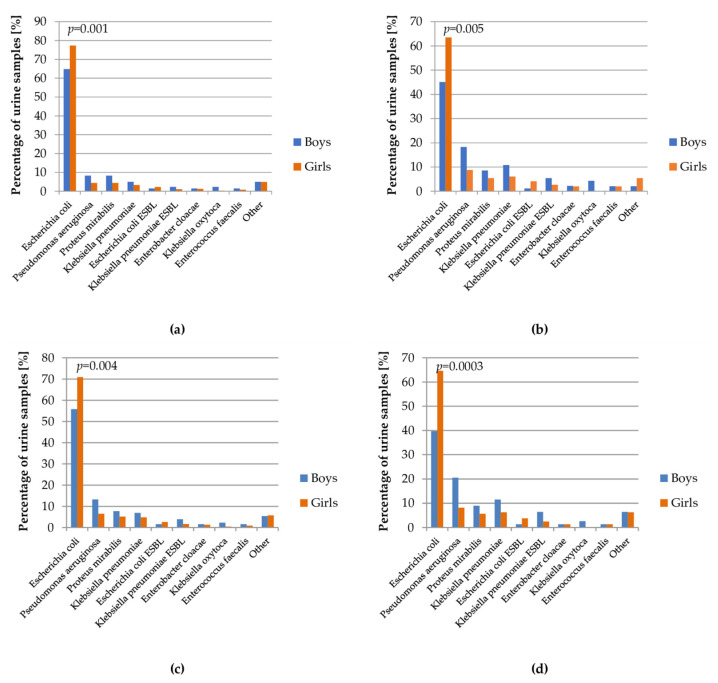
Percentage of bacterial strains in the urine cultures depending on gender of patients: (**a**) total; (**b**) patients with urinary tract defect; (**c**) patients on chemoprophylaxis; (**d**) patients with recurrent UTI.

In the performed analysis, there was a lower percentage of urine samples with *Escherichia coli* in boys than in girls (64.8% vs. 77.3% in whole examined group), similar to the ESBL strain of this bacteria (1.4% vs. 2.2% in whole examined group). Other microbes accounted for a greater percentage in males than females (Figure 4).

**Figure 5:**
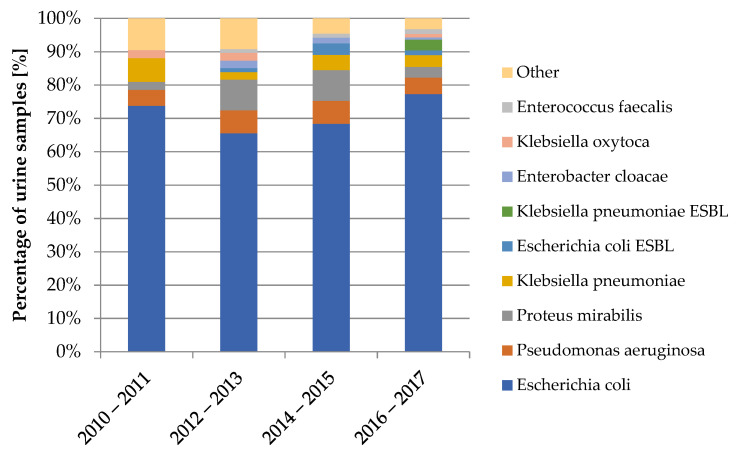
Variability of bacterial strains in the urine cultures from 2010–2017.

The diagram in Figure 5 shows the variability of bacterial strains isolated from urine samples over the years covered by the study.

**Figure 6:**
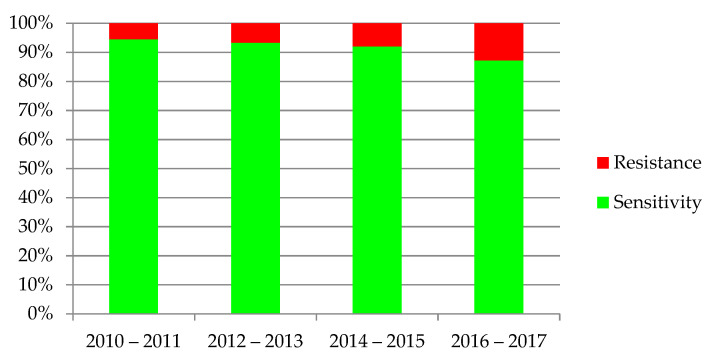
Sensitivity and resistance of bacteria to antibiotics from 2010–2017.

The increasing resistance of bacteria was observed in antibiograms of the urine cultures, shown in Figure 6. Dependency was statistically significant (*p* < 0.001).

**Figure 7:**
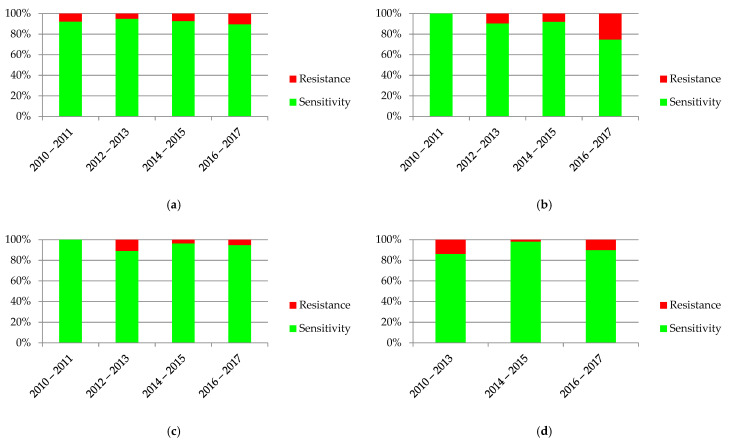
Sensitivity and resistance of bacteria to antibiotics from 2010–2017: (**a**) *Escherichia coli*; (**b**) *Pseudomonas aeruginosa*; (**c**) *Klebsiella pneumonia*; (**d**) *Proteus mirabilis*.

The diagrams in Figure 7 show the variability over time in antibiotic resistance of the most common bacteria. Statistical significance was found in the case of *E. coli* (*p* = 0.0002), *K. pneumoniae* (*p* = 0.004) and *P.* *mirabilis* (*p* = 0.006).

**Figure 8:**
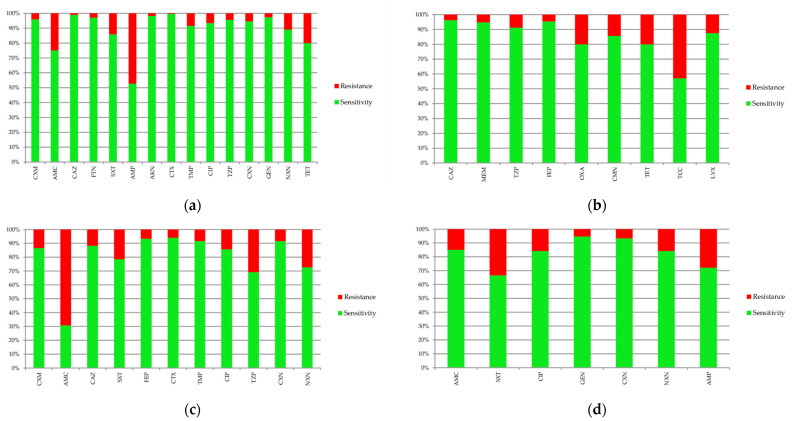
Sensitivity and resistance of bacteria to antibiotics: (**a**) *Escherichia coli*; (**b**) *Pseudomonas aeruginosa*; (**c**) *Klebsiella pneumonia*; (**d**) *Proteus mirabilis*. AKN—amikacin, AMC—amoxicillin with clavulanic acid, AMP—ampicillin, CAZ—ceftazidime, CFM—cefixime, CIP—ciprofloxacin, CMN—clindamycin, CTX—cefotaxime, CXM—cefuroxime, CXN—cephalexin, FEP—cefepime, FTN—nitrofurantoin, GEN—gentamicin, LVX—levofloxacin, MEM—meropenem, NXN—norfloxacin, OXA—oxacillin, SXT—cotrimoxazole, TCC—ticarcillin with clavulanic acid, TET—tetracycline, TMP—trimethoprim TZP—piperacillin with tazobactam.

Comparisons of sensitivity of the most dominant bacteria to antibiotics are presented in Figure 8. In the case of *Escherichia coli*, the highest percentage of resistance was found in ampicillin (47.2%) and amoxicillin with clavulanic acid (24.8%). *Pseudomonas aeruginosa* was characterized by a high percentage of resistance to ticarcillin with clavulanic acid (42.9%). It is necessary to mention that sensitivity to this antibiotic was estimated on the basis of only seven antibiograms. *Klebsiella pneumoniae* was most resistant to amoxicillin with clavulanic acid (69.2%); however, *Proteus mirabilis* was most resistant to cotrimoxazole (33.3%).

**Figure 9:**
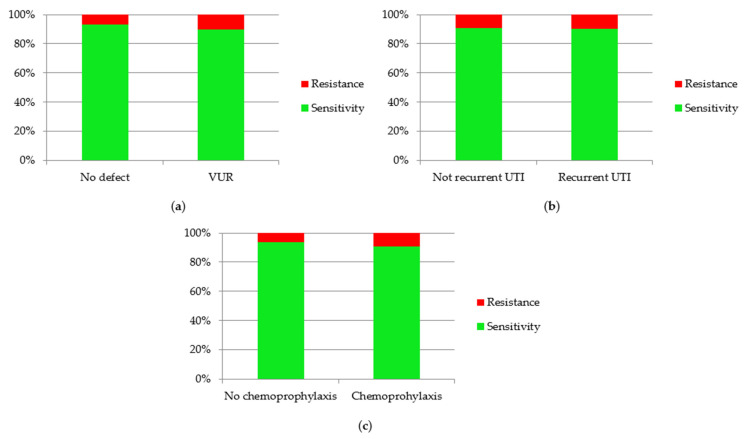
Sensitivity and resistance of *Escherichia coli* to antibiotics in children with: (**a**) VUR; (**b**) recurrent UTI; (**c**) chemoprophylaxis.

Considering risk factors of UTI, such as VUR and recurrence of infections, the association between the presence of them and antibiotic resistance was assessed (Figure 9). Only episodes of UTI caused by *E. coli* were analyzed. Other etiological factors were not assessed due to the insufficient number of cases, which did not allow for obtaining reliable results. The percentage of antibiotic resistance were higher in UTI episodes in children with VUR (*p* = 0.002). Antibiotic resistance was observed a little more often in cases of recurrent UTI (9.93%) than in non-recurrent UTI (9.04%), but there was no statistical significance (*p* = 0.37). Additionally, assessment of antibiotic resistance in children on chemoprophylaxis was performed. Resistant *E. coli* was observed more often in children on chemoprophylaxis (*p* < 0.001).

**Figure 10:**
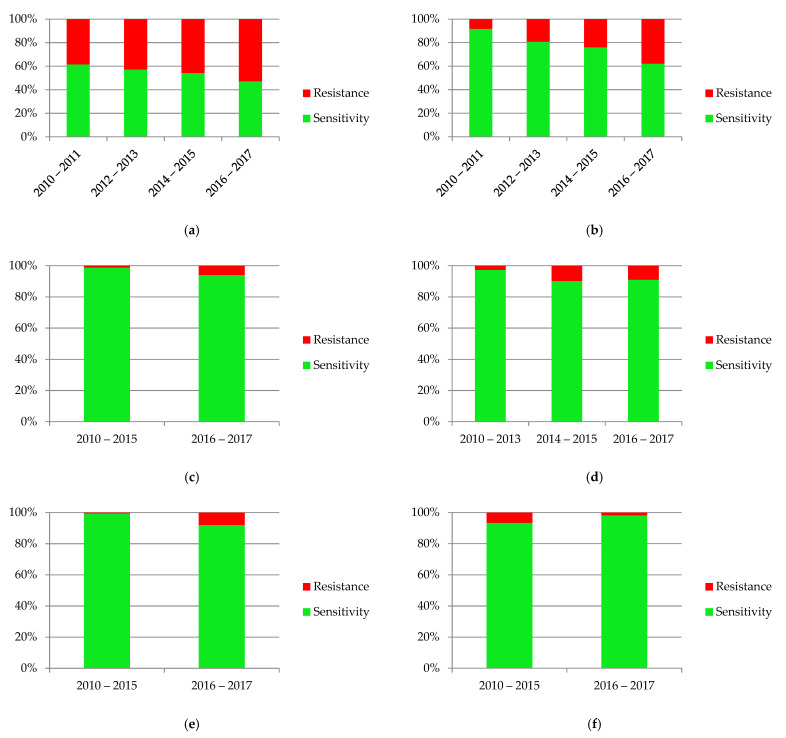

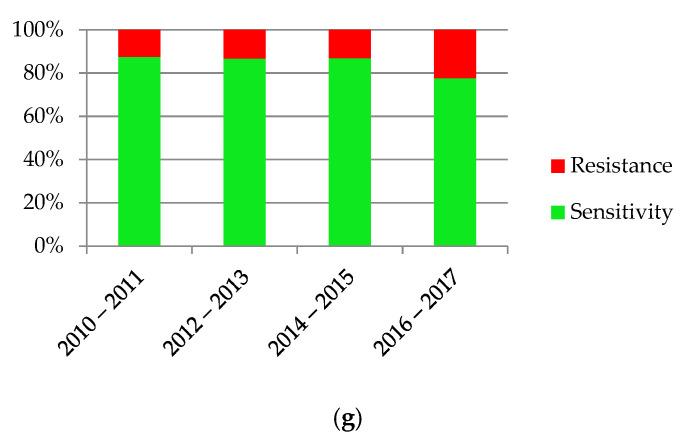
Sensitivity and resistance of bacteria to antibiotics from 2010–2017: (**a**) ampicillin; (**b**) amoxicillin with clavulanic acid; (**c**) gentamicin; (**d**) cefuroxime; (**e**) ceftazidime; (**f**) nitrofurantoin; (**g**) cotrimoxazole.

The diagrams in Figure 10 present the variability of sensitivity to most commonly used antibiotics. There was a significant increase of resistance to amoxicillin with clavulanic acid (*p* = 0.001), gentamicin (*p* = 0.017) and ceftazidime (*p* = 0.0005). The only drug where decreasing resistance was observed was nitrofurantoin (*p* = 0.032).

Further tests were carried out to identify differences in values of inflammatory markers between resistant and sensitive *Escherichia coli* to commonly used antibiotics. In the analysis, there were found to be significantly lower values of WBC in patients with *Escherichia coli* resistant to ampicillin (*p* = 0.04), cefuroxime (*p* = 0.007) and nitrofurantoin (*p* = 0.0045) beside sensitive ones. In the case of CRP, higher concentrations were observed in participants with *E. coli* sensitive to cefuroxime (*p* = 0.045) and nitrofurantoin (*p* = 0.02). There was no significant difference in analyzed values of PCT in the case of any antibiotic. It was not possible to evaluate statistical significance of PCT difference for nitrofurantoin because of no PCT test in patients with *E. coli* resistance to this antibiotic. However, for ceftazidime, too few resistant samples to carry out the analysis were observed.

**Figure 11:**
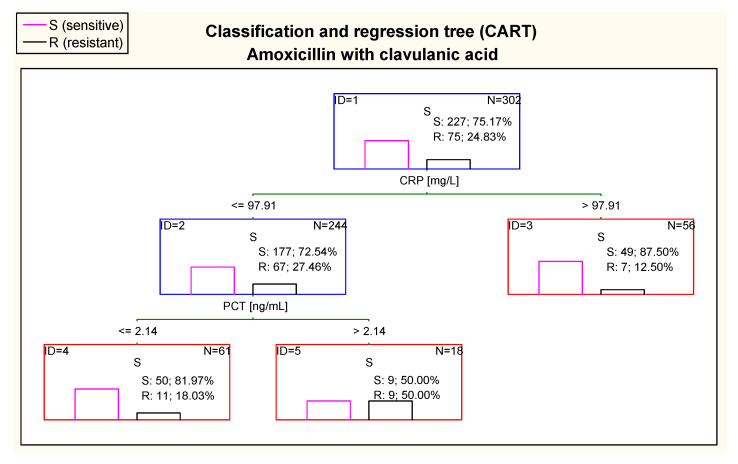
Classification and regression tree (CART) for resistance of *Escherichia coli* to amoxicillin with clavulanic acid created on basis of inflammatory markers.

Taking into consideration resistance to amoxicillin with clavulanic acid, the CART tree showed differences in CRP and PCT concentrations (Figure 11). In children with CRP value > 97.91 mg/L, there was great percentage of sensitive cases (87.5%), while CRP ≤ 97.91 mg/L was associated with higher risk of smaller sensitivity (72.54%). However, in cases with CRP ≤ 97.91 mg/L and PCT ≤ 2.14 ng/mL there was greater chance to have sensitivity to amoxicillin with clavulanic acid (81.97% of children) than in the case of PCT > 2.14 ng/mL.

**Figure 12:**
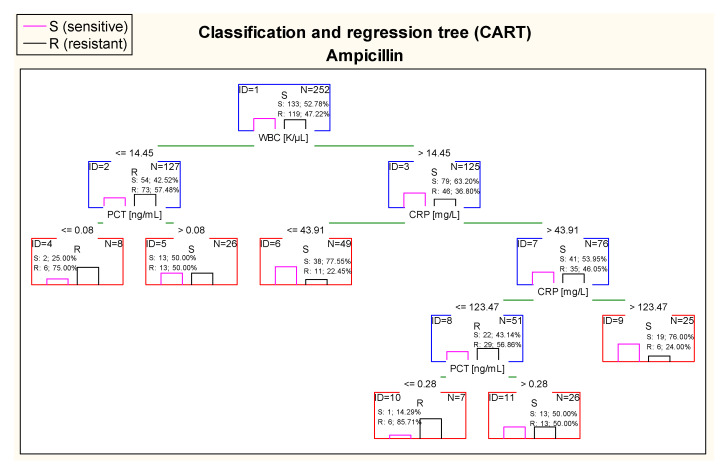
Classification and regression tree (CART) for resistance of *Escherichia coli* to ampicillin created on the basis of inflammatory markers.

Figure 12 shows that the most optimal cut–off point of WBC in case of ampicillin in the first node of the CART was 14.45 K/µL. Children with values of WBC above 14.45 K/µL were classified as patients with *E. coli* expected to be more sensitive to ampicillin. On the other hand, CRP in the CART was also taken into consideration. In participants with WBC > 14.45 K/µL, patients with CRP in the range of 43.91–123.47 mg/L were classified as patients with higher risk of resistance to ampicillin than children with CRP > 123.47 mg/L. Considering PCT concentration in this tree, children with WBC below or equal 14.45 K/µland with PCT ≤ 0.08 ng/mL were at high risk of resistance to ampicillin (75.0% of samples). However, it is important to notice that there were only eight cases in this tree node. A similar situation took place in children with CRP values between 43.91 mg/L and 123.47 mg/L; patients with a concentration of PCT ≤ 0.28 ng/mL were at higher risk of resistance to ampicillin (85.71% of children). However, this tree node was also presented with small number of cases.

**Figure 13:**
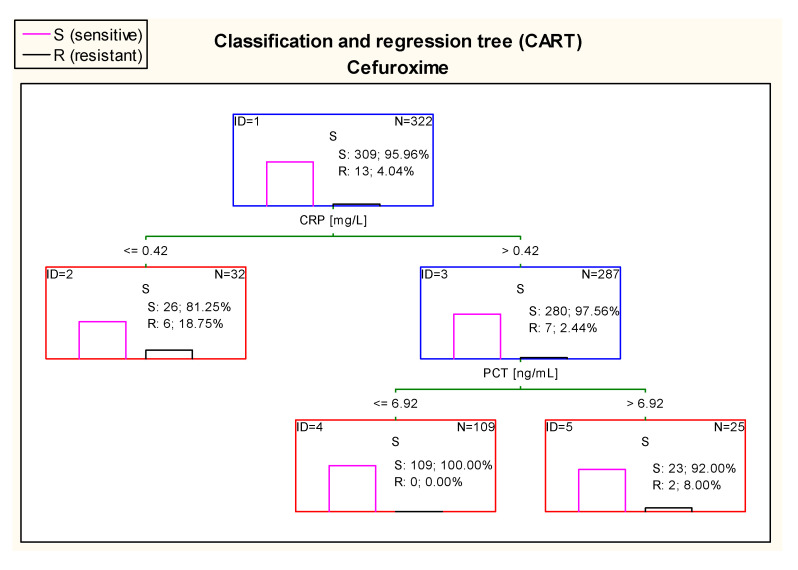
Classification and regression tree (CART) for resistance of *Escherichia coli* to cefuroxime created on the basis of inflammatory markers.

Further, in case of cefuroxime, there were optimal cut–off points of CRP and PCT values in estimating risk of resistance of *E.* *coli*. 100% of children with CRP > 0.42 mg/land PCT ≤ 6.92 ng/mL had confirmed sensitivity to cefuroxime in antibiograms (Figure 13).

**Figure 14:**
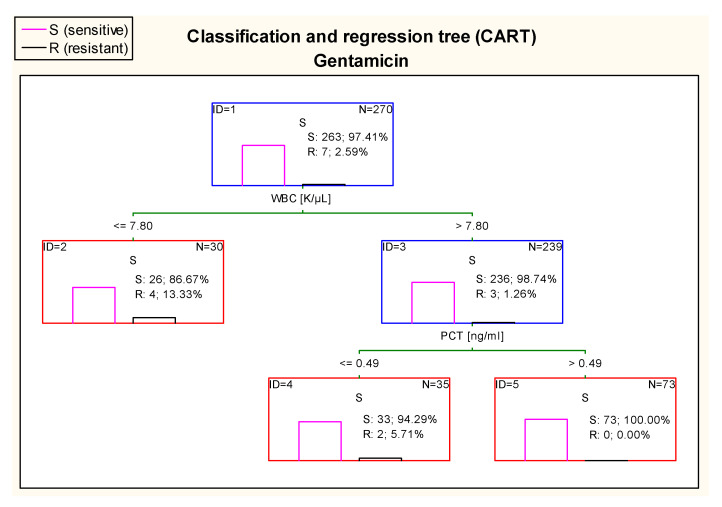
Classification and regression tree (CART) for resistance of *Escherichia coli* to gentamicin created on the basis of inflammatory markers.

The CART tree in Figure 14, concerning sensitivity to gentamicin, sets the most optimal cut-off point of WBC at the level of 7.80 K/µL. Additionally, 100% of *E. coli* isolated from urine samples of patients with WBC > 7.80 K/µL and PCT concentration > 0.49 ng/mL were sensitive to this drug.

**Figure 15:**
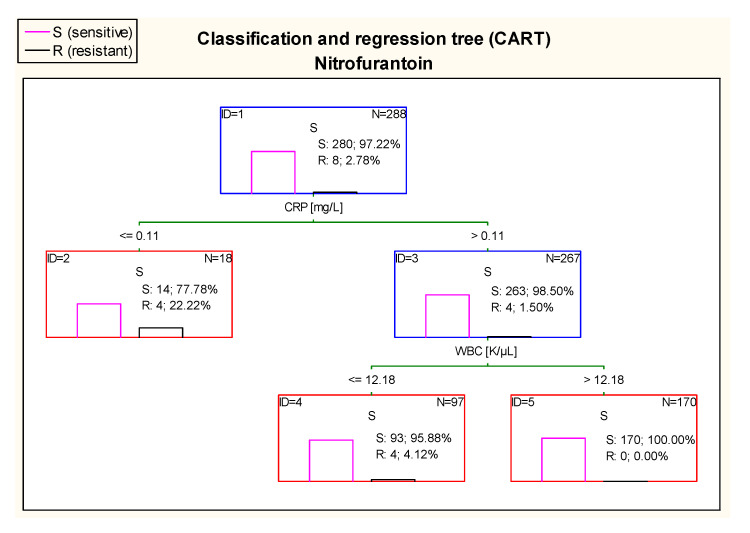
Classification and regression tree (CART) for resistance of *Escherichia coli* to nitrofurantoin created on the basis of inflammatory markers.

The diagram in Figure 15 shows that 100% of patients who had UTI caused by *E. coli* sensitive to nitrofurantoin had CRP > 0.11 mg/L and WBC value higher than 12.18 K/µL.

## 4. Discussion

Infections of the urinary tract in children are of great importance in epidemiology and clinical practice. They account for a large percentage of bacterial infections in the pediatric population. The proper diagnosis and treatment reduce the frequency of observed complications, such as chronic kidney disease or hypertension. Moreover, rationally conducted antibiotic therapy helps to inhibit the growing resistance of bacteria to drugs.

In the present study, we undertook the assessment of epidemiology and antibiotic resistance patterns of pathogens in accordance with inflammatory parameters. Research included 525 children aged between 1 week and 18 years old, in which there were observed 627 episodes of UTI. In prior research studies conducted in pediatric patients, more than 75% of children under 5 years old with febrile UTI showed features of pyelonephritis in renal scintigraphy [18,19], which in 27–64% led to kidney scarring [20,21]. Winberg et al. [22] estimated that up to the beginning of puberty, UTI was diagnosed in 1% of boys and 3% of girls, while a study by Stark [23], conducted in 1997 showed a higher percentage of UTI in prepubertal girls (8%). According to NICE (National Institute for Health and Care Excellence), 1/10 females and 1/30 males will have developed a UTI by the age of 16 [24]. In the study by Milas et al. [25], on the group of 1200 newborns, UTI was diagnosed in 4.5% of patients. Data from literature shows the highest percentage of infections among children < 1 year old [26,27]. According to the research work of Hanna–Wakim et al. [28], the median age of diagnosis of UTI was 16 months. Numerous prior studies about dependence of gender on UTI incidence unanimously indicate the superiority of diagnoses in boys in the first 2–3 months of life and the increase of incidence of UTI in females after the first year of life [29,30,31,32,33]. In our study, only children hospitalized at the Department of Pediatrics and Nephrology were assessed; therefore, it was impossible to estimate the overall incidence of UTI in the pediatric population. Our research showed that 38.6% of UTI episodes occurred in patients < 1 year old and infections in children < 2 years old accounted for 48.8% of all cases. The median age of UTI onset (25 months) was slightly higher than in Wakim’s et al. research [28]. In the analysis, a significant relationship between gender and age of patients was found, confirming a prevalence of UTI episodes in boys < 6 months old and an increase of UTI incidence in girls after the second year of life.

Initially, we analyzed individual parameters of patients, including risk factors. One of them is a history of infections. In our study, we found that about one third of girls have a history of recurrent UTIs, while in boys this percentage was about 23%. Literature data shows that recurrent UTIs are observed in 13–33% of children with a predominance of females [22,34,35,36,37]. Herein, obtained results are consistent with prior studies.

The other risk factors, which were included in our analysis, were anatomical and functional disorders of the urinary tract. The most common abnormality is VUR. In our study, VUR was diagnosed in 18.4% of girls and in 16% of boys. There was a correlation between grade of VUR and gender of patients–boys were statistically more likely to have high-grade VUR than girls (*p* < 0.05). In the study by Orellana et al. [38], among 269 pediatric patients diagnosed with ≥1 UTI episodes, 55.8% of children had VUR (within 234 renal units); in the case of 101 renal units, it was a high-grade VUR. This defect was more common in children < 1 year old (*p* = 0.001) and, contrary to our results, in boys. In the research study by Swerkersson et al. [39], VUR were observed in 22% of boys and in 31% of girls. Interestingly, the high–grade VUR was found significantly more often in boys (*p* < 0.01), what was also obtained in our study.

The next step in our study was to assess the variability of bacterial strains in urine cultures and their susceptibility to antibiotics. Due to actual guidelines, the positive urine culture (with significant bacteriuria) and the presence of leukocyturia in urinalysis are necessary to reach a UTI diagnosis. In our analysis, pyuria was found in 78.8% of urine samples. In the remaining 21.2% of samples, in 34 cases there was outpatient antibiotic therapy administered (5.4% of all). In most of the remaining cases, lack of leukocyturia was concerned in infants and children < 2 years old who hadyet to complete toilet training. Therefore, urine is not kept in the bladder long enough to detect pyuria in the urinalysis in children at this age. Kim et al. [40] conducted the study to find out if absence of leukocyturia can exclude UTI in febrile children < 24 months of age. They observed that percentage of features of pyelonephritis in renal scintigraphy in both groups (patients with fulfilled diagnostic criteria of UTI and patients with clinical, even nonspecific, symptoms of UTI) were comparable (47.8% vs. 50.0%). Moreover, Shaikh et al. [41], estimated that about 12 children with UTI without pyuria were being overlooked to protect one patient with asymptomatic bacteriuria from antibiotic administration. Due to these studies and our analysis, it seems to be reasonable to diagnose UTI by considering all the components of clinical picture.

In recent years, the variability of microorganisms detected in urine cultures has increased. Considering the prior studies, the most dominant bacterial strain is still *Escherichia coli*, which varies between 53% and 87% [10,42,43,44,45]. The other microbes found in urine cultures were *Klebsiella pneumoniae* (7–36.2%), *Proteus mirabilis* (3.0–3.49%) and *Pseudomonas aeruginosa* (2.3–7.57%). Our study also identified *E. coli* as the most dominant UTI etiological factor (72.7%). Contrary to presented studies, we observed the lower percentage in UTIs caused by *K. pneumoniae* (3.9%) and greater number of cases with *P. mirabilis* etiology (5.8%), which may be correlated with no tradition of boys’ circumcising in Poland and consequently with the presence of foreskin. The same studies took into consideration variability of antibiotic susceptibility. The antibiotic with the greatest percentage of resistance was ampicillin (83–87%). High-grade resistance was also found in ceftriaxone (40–62%), cotrimoxazole (40–93%) and amoxicillin with clavulanic acid (30–83%) [10,42,43,44,45]. Another Polish center (The Department of Medical Microbiology, Medical University of Silesia, Poland) carried out a research study on 710 urine samples, which showed high sensitivity of *E. coli* to gentamicin (97.7%) or amikacin (96.0%) and high resistance to ampicillin (70%) [46]. Another study was conducted in Granada (Spain) among <2–year–old patients, where Sorlózano-Puerto et al. [47], found that *E. coli* was resistant to amoxicillin with clavulanic acid and cotrimoxazole in about 20–30% of samples (depend on age group). These results showed similarity with our outcomes.

In our research, the percentage of resistance to commonly used antibiotics was lower than in prior studies, although we observed significantly decreasing susceptibility of bacteria. In the case of *E. coli*, the highest resistance was found against ampicillin (47.2%) and amoxicillin with clavulanic acid (24.8%). Moreover, our results were interesting with regard to obtained statistical significance in increasing antibiotics resistance to amoxicillin with clavulanic acid (*p* < 0.01), gentamicin (*p* < 0.05) and ceftazidime (*p* < 0.01). Interestingly, the increasing sensitivity to nitrofurantoin was observed (*p* < 0.05). Prior studies and our results confirmed an unwanted trend in increasing antibiotic resistance. In addition, this phenomenon is observed in relation to drugs used in severe infections, such as meningitis, which makes it even more alarming. On the other hand, an increasing sensitivity to nitrofurantoin may indicate more frequent use of antibiotics instead of chemotherapeutics in mild infections. Furthermore, the decreasing susceptibility to commonly used antibiotics, such as amoxicillin with clavulanic acid, is worth mentioning due to its misuse (e.g., in viral infections).

Finally, we tried to assess whether the inflammatory markers are associated with susceptibility of microbes to used antibiotics. In the available literature, we did not find any information about research studies related to this topic. Unfortunately, we were unabl to estimate precise values of CRP, PCT or WBC at which the antibiotic resistance can be ascertained. However, by using the CART trees method, we were able to slightly determine inflammatory markers’ values to which we can suspect that bacteria are predisposed to be resistant to antibacterial drugs.

In summary, we made an attempt to evaluate cut-off points of inflammatory markers’ values in case of amoxicillin with clavulanic acid, ampicillin, cefuroxime, gentamicin and nitrofurantoin (despite increasing antibiotic resistance, ampicillin and amoxicillin with clavulanic acid are still widely used). Considering amoxicillin with clavulanic acid, this point was determined at the value of CRP of 97.91 mg/L, above which there were observed higher percentages of sensitive cases. The high risk of resistance to ampicillin was found in children with WBC below or equal 14.45 K/µL and PCT ≤ 0.08 ng/mL. In the case of WBC > 14.45 K/µL, the resistance’s risk was greater if CRP was in the range of 43.91–123.47 mg/L and PCT was ≤0.28 ng/mL. The most important results concerning cefuroxime revealed that all patients with values of CRP > 0.42 mg/L and PCT ≤ 6.92 ng/mL had confirmed susceptibility. Moreover, our results were interesting with regard to estimate the cut-off point for gentamicin—100% of *E. coli* isolated from children with WBC above 7.80 K/µL and PCT value > 0.49 ng/mL were sensitive. In case of nitrofurantoin, we found that values of CRP above 0.11 mg/L and WBC above 12.18 K/µL were associated with susceptibility to this drug (100% of cases). Results of our study seem to be useful due to the fact that urine culture is a time consuming method. There is a chance to initially assess whether the bacterium will be sensitive to used antibiotics and predict empiric antimicrobial treatment if we know the values of inflammatory markers, until urine cultures become available.

There were some limitations to this study, including that it was small, single–center and contained regional data. We have also focused on the one specific infection that could have had an impact on the calculated sensitivity of the inflammatory markers, which should therefore be interpreted with caution. Furthermore, there could have been clinical signs of severity in some patients that were not captured in our study and that would justify the use of antibiotics. Finally, we described the added value of markers of inflammation testing to guide antibiotic prescription without considering patient–related factors, such as the uptake of prescribed antibiotics and acceptance of a non–antibiotic management strategy by the patient. Not all clinical variables that may affect inflammatory markers, such as anaemia, were included in the evaluation.

Reached results indicate that increasing antibiotic resistance is a significant ongoing problem. Due to high percentage of resistance to amoxicillin with clavulanic acid, it is reasonable not to use this antibiotic empirically in our region. However, cefuroxime seems to be a promising choice as a first-line treatment. Our initial study results show that there are potential factors such as specific CRP and WBC levels, which can help in making decisions of first-line empiric antibiotic therapy. However, more studies on a larger group of patients are needed do confirm these data.

## Figures and Tables

**Table 1:** Demographic, anthropometric and biochemical parameters in patients; comparisons between males and females.

		*n*	Median	Q1	Q3	*p*-Value
Age (years)	all	627	2.08	0.5	8.67	
	male	229	0.58	0.25	4.75	<0.001
	female	398	3.88	0.92	10.42	
Body weight (kg)	all	600	12.00	7.40	26.00	
	male	219	8.70	6.20	15.00	<0.001
	female	381	16.40	9.10	31.00	
BMI (kg/m^2^)	all	264	17.09	15.09	19.96	
	male	50	16.50	14.89	18.27	NS
	female	214	17.17	15.09	20.66	
CRP (mg/L)	all	622	14.93	1.56	66.38	
	male	229	14.95	1.78	62.90	NS
	female	393	14.90	1.52	70.00	
PCT (ng/mL)	all	228	0.65	0.15	4.16	
	male	95	0.75	0.11	6.70	NS
	female	133	0.60	0.16	3.46	
WBC (K/µL)	all	624	12.60	9.10	17.70	
	male	229	13.59	9.90	18.70	<0.01
	female	395	11.90	8.80	17.20	

Q1—lower quartile; Q3—upper quartile; NS—not significant.

**Table 2:** Anatomical defects of urinary tract depending on gender of patients.

Type of Anatomical Defect ^1^		Male (*n* = 194)	Female (*n* = 331)	Total (*n* = 525)
Duplex collecting system	*n*	8	12	20
	[%]	4.12%	3.63%	3.81%
Ureteropelvic junction obstruction	*n*	6	8	14
	[%]	3.09%	2.42%	2.67%
Ureterovesical junction obstruction	*n*	5	4	9
	[%]	2.58%	1.21%	1.71%
Vesicoureteral reflux	*n*	31	61	92
	[%]	15.98%	18.43%	17.52%
Urethral valves	*n*	18	-	-
	[%]	9.28%	-	-

^1^ Some patients had more than 1 type of anatomical defect.

**Table 3:** Number of children with VUR depending on gender.

VUR		Male	Female	Total	
VUR I–III	*n*	16	47	63	*p* = 0.013
	[%]	51.6%	75.4%	68.5%	
VUR IV–V	*n*	15	14	29	
	[%]	48.4%	24.6%	31.5%	
Total	*n*	61	31	9	

## Data Availability

The datasets used and/or analyzed during the current study are available from the corresponding author on reasonable request.
